# The Impact of Moyamoya Disease and *RNF213* Mutations on the Spectrum of Plasma Protein and MicroRNA

**DOI:** 10.3390/jcm8101648

**Published:** 2019-10-10

**Authors:** Ming-Jen Lee, Shannon Fallen, Yong Zhou, David Baxter, Kelsey Scherler, Meng-Fai Kuo, Kai Wang

**Affiliations:** 1 Department of Neurology, National Taiwan University Hospital, Taipei 10002, Taiwan; mjlee@ntu.edu.tw; 2 Institute for Systems Biology, Seattle, WA 98109, USA; sfallen@systemsbiology.org (S.F.); yong.zhou@systemsbiology.org (Y.Z.); dbaxter@systemsbiology.org (D.B.); kscherler@systemsbiology.org (K.S.); 3 Department of Neurosurgery, National Taiwan University Hospital, Taipei 10002, Taiwan; mfkenator@gmail.com

**Keywords:** moyamoya disease, extracellular vesicle, RNAseq, biomarker, cerebrovascular disorder

## Abstract

Moyamoya disease (MMD) is a rare cerebrovascular disorder characterized by occlusion of bilateral internal carotid and intracerebral arteries with the compensatory growth of fragile small vessels. MMD patients develop recurrent infarctions in the basal ganglia and subcortical regions. Symptoms include transient ischemic attack or stroke, seizures, and headaches, which may occur suddenly or in a stepwise progression. Mutations in Ring Finger Protein 213 (*RNF213*), a Zinc ring finger protein, have been identified in some MMD patients but the etiology of MMD is still largely unknown. To gain insight into the pathophysiology of MMD, we characterized the impact of the *RNF213* mutations on plasma protein and RNA profiles. Isobaric tags for relative and absolute quantitation and proximity extension assay were used to characterize the plasma proteome. Next generation sequencing-based small RNAseq was used to analyze the cell-free small RNAs in whole plasma and RNA encapsulated in extracellular vesicles. The changes of miRNAs and proteins identified are associated with signaling processes including angiogenesis and immune activities which may reflect the pathology and progression of MMD.

## 1. Introduction

Moyamoya disease (MMD) is a rare disorder first described in 1969 in Japanese patients [1] and the majority of cases are from Eastern Asian descent. MMD is characterized by bilateral stenosis of internal carotid arteries (ICAs) with frequent involvement of anterior and middle cerebral arteries. The stenotic ICAs in MMD patients show thickening of intima, proliferation of smooth muscle cells, and prominently tortuous and duplicated internal elastic lamina [2,3]. To compensate for the occlusion, a large number of tiny and weak vessels are overgrown with the appearance of a “puff of smoke” (termed moyamoya in Japanese) under conventional angiography. There are two major categories of symptoms: ischemic attacks (stroke, reversible ischemic neurological deficits, and seizure) and the deleterious consequences of the compensatory mechanism due to ischemia (hemorrhage from the fragile collateral vessels and headache from dilated transdural collaterals) [4,5]. Several genetic loci have been linked to MMD and mutations in *RNF213* [6,7] and *ACTA2* [8] genes have been identified in some patients, but the etiology of disease is still unclear. 

Since the MMD pathologies are associated with blood vessels, characterizing the molecular changes of plasma in patients with MMD may yield insights into the disease. For example, the process of compensatory revascularization is associated with increased inflammatory signals and angiogenic factors in blood including hypoxia-inducible factor-1, vascular endothelial growth factor (VEGF), fibroblast growth factor (FGF) 2, transforming growth factors, matrix metalloproteinases (MMPs), and granulocyte-macrophage colony-stimulating factor (GM-CSF, CSF2) [9,10,11,12,13,14]. 

In addition to proteins, blood also contains cell-free RNAs, especially microRNA (miRNA). Recently, cell-free miRNA, a class of short noncoding regulatory RNA, have been harnessed as biomarkers for various physiopathological conditions. For example, miR-122 levels in circulation are an indicator for liver diseases [15], and concentration changes of miR-208 and miR-499 are associated with heart conditions [16,17]. These cell-free circulating miRNAs are either bound to RNA binding proteins, such as Nucleophosmin 1 (NPM1) or Argonaute 2 (Ago2) [18,19], or lipoproteins, such as high-density lipoprotein (HDL) or low density lipoprotein (LDL) [20], or encapsulated into extracellular vesicles (EVs) to evade RNase degradation [21,22]. EVs in circulation may play a role in cell–cell communication [23]. Therefore, characterizing the molecular content in EVs is of great interest.

Here, we employ isobaric tags for relative and absolute quantitation (iTRAQ), a global proteome profiling approach and a modified enzyme-linked immunosorbent assay (ELISA) called proximity extension assay (PEA) technology [24] (Olink proteomics, Uppsala, Sweden) to characterize the impact of MMD on plasma proteome. In addition, we used an in-house developed small RNA library construction protocol to characterize the cell-free miRNAs, in whole plasma, EVs (miRNA inside of EVs) and EV-depleted plasma (miRNA outside of EVs) that may provide insights into the perturbed molecular processes involved in MMD. Comparing the miRNA profiles between EVs and EV-depleted plasma also allowed us to determine the distribution of specific miRNA between in and outside of EVs. To our knowledge, this is the first comprehensive characterization of circulating miRNA and proteins, as well the distribution of circulating miRNA inside and outside of EVs in MMD patients.

## 2. Materials and Methods

### 2.1. Ethics Statement and Patient Information

This study was approved by the Research Ethics Board of National Taiwan University Hospital (201506040RINB) and conducted according to the principles of the Declaration of Helsinki. Blood samples were collected from patients who had been diagnosed with MMD and healthy controls. All patients provided written informed consent to participate in this study. The information of study participants is listed in Table S1.

This study included 7 MMD patients who carry a mutated *RNF213* (MMD/*RNF213*+), 5 patients without *RNF213* mutation (MMD/*RNF213*-), and 12 controls. The clinical presentation of these patients has been described previously [25]. The controls included 6 parents of MMD patients and 6 siblings.

### 2.2. Plasma Preparation and Extracellular Vesicle (EV) Isolation

MMD patient blood was collected from patients into K_2_-EDTA blood collection tubes after the EDAS surgery. The control blood samples were collected during the patient’s first clinic visit. The blood was centrifuged at 1000× *g* for 10 min at 4 °C and the supernatant (plasma) was transferred to a new tube and centrifuged at 2500× *g* for 15 min. The plasma was then aliquoted into smaller polypropylene tubes and stored at −80 °C. Prior to EV isolation or RNA extraction, plasma was spun at 10,000× *g* for 15 min at 4 °C. EVs were isolated from 200 μL of plasma using size exclusion chromatography (SEC) columns (iZON qEV, Cambridge, MA, USA) with de-gassed 1× PBS (pH 7.2, Gibco, Grand Island, NY, USA). The protocol for EVs and EV-depleted plasma preparation was described previously [26]. 

### 2.3. Isolation of RNA and Small RNA Sequencing Library Construction 

RNA was isolated from plasma, corresponding EVs and EV-depleted plasma samples using miRNeasy Micro Kit (Qiagen, Germantown, MD, USA). The quality and quantity of the RNA were evaluated with the Agilent 2100 Bioanalyzer (Santa Clara, CA, USA) and NanoDrop 1000 spectrophotometer (Thermo Scientific, Wilmington, DE, USA). Small RNA sequencing libraries were generated using a modified library construction protocol [27]. Modifications include adding four random nucleotides at the appropriate end of the adapters to reduce ligation-associated bias, the use of higher adapter concentrations, and increased amount of polyethylene glycol in the ligation steps. In addition, two rounds of size selection steps are performed, following each amplification step, to reduce the adapter dimer. Individual library concentrations were measured using the NEBNext Library Quant Kit (New England Biolabs, Ipswich, MA, USA) and pooled to a final concentration of 2 nM then sequenced using the NEXTseq DNA sequencer (Illumina, San Diego, CA, USA).

### 2.4. Small RNAseq Data Analysis and Verification 

The sequencing results were analyzed using sRNAnalyzer (http://srnanalyzer.systemsbiology.net/) [28], which contains three major components: pre-processing, mapping, and results summarization. In the pre-processing step, the adaptor sequences used in the library construction were trimmed and the low-quality sequences were removed. The processed sequences were then mapped against various databases. A detectable miRNA species is defined as having at least 10 mapped reads with no mismatch allowed in at least 50% of the samples.

Taqman miRNA Assays were used to verify the concentration changes of specific miRNA identified by next generation sequencing (NGS). In brief, RNA (2 μL) from individual samples was reverse transcribed with the TaqMan microRNA RT kit (Thermo Fisher, Waltham, MA, USA). Real time qPCR was performed using the BioRad CFX96 Touch real-time thermocycler (BioRad, Hercules, CA, USA) with the following condition: 95 °C for 10 min followed by 40 cycles of amplification at 95 °C for 15 s and 60 °C for 60 s. Hsa-miR-16-5p was used to normalize the results due to its high concentration and low variation in plasma.

### 2.5. Plasma Protein Analysis

Global plasma proteome changes associated with MMD were measured using isobaric tags for relative and absolute quantitation (iTRAQ) (Table S1). In brief, the 14 most abundant plasma proteins were removed with a human Mars14 LC column (Agilent, CA, USA). The flow-through fractions of each sample were collected and concentrated, and the protein concentrations were determined by bicinchoninic acid (BCA) assay (Pierce, Rockford, IL, USA). Proteins were digested with trypsin following a 2,2,2-Trifluoroethanol (TFE)-denaturation and desalted on Oasis C18 cartridges (Waters, Milford, MA, USA) before iTRAQ labeling. Approximately 60 µg of tryptic peptides from each sample was labeled with one of the eight isobaric isotopic labeling reagents (8-plex iTRAQ, tag m/z 113, 114, 115, 116, 117, 118, 119 and 121 (AB SCIEX, Framingham, MA, USA) on N-termini and lysine residues. Samples were combined and fractionated using a high-pH reverse-phase HPLC into 12 fractions. iTRAQ-labeled samples were analyzed on a Q Exactive™ Plus Hybrid Quadrupole-Orbitrap mass spectrometer. Acquired data were processed in Trans Proteomic Pipeline (TPP) (V4.8) including PeptideProphet and ProteinProphet. The data were searched using the Open Mass Spectrometry Search Algorithm (OMSSA) [29] against an International Protein Index (IPI) human database (version 3.87) with the addition of decoy sequences. Libra, an in-house script, was performed to extract ratios of isobaric tags in MS/MS spectra. The threshold of minimum intensity for isobaric tags was set at 20. Mass tolerance was 0.1 Da. All ratios were normalized to one common channel in both iTRAQ runs. Only proteins with more than 2 unique peptides identified and quantified in all channels were included. Ratios for each protein were reported as significantly different if they showed a more than 50% change of concentration (vs. average of normal controls) with a *p*-value of <0.05 from a two-tailed student’s *t*-test. 

A panel of 92 immune response-related proteins (Table S2) were measured using PEA following the manufacturer’s protocol (Olink Proteomics, Uppsala, Sweden). Briefly, 1 μL of plasma sample was added to each well of an incubation plate containing 3 μL of incubation mix and then incubate the plate at 4 °C overnight to allow antibody to bind with its analyte. The following day, 96 μL of an extension mix was added to each well of the incubation plate and, within five minutes, transferred to a preprogrammed thermocycler for the extension step. For the detection step, 5 μL of each primer (provided on a primer plate) was loaded onto a primed 96 by 96 Dynamic Array Integrated Fluidic Circuit (IFC) from Fluidigm (South San Francisco, CA, USA). Lastly, 2.8 μL of the extension products were combined with 7.2 μL of detection mix and 5 μL was loaded onto the primed chip. The chip was loaded in the Fluidigm IFC Controller HX and the Olink Protein Expression 96 × 96 Program was run on the Fluidigm’s Biomark HD System.

### 2.6. Functional Enrichment and Network Analyses

The biological impacts of MMD-associated circulating miRNAs were assessed by using validated miRNA–mRNA interactions from miRTarBase database (mirtarbase.mbc.nctu.edu.tw) [30,31] instead of predicted miRNA–mRNA integrations. Functional enrichment analyses were conducted with Database for Annotation, Visualization, and Integrated Discovery (DAVID) [32]. The Kyoto Encyclopedia of Genes and Genomes (KEGG) pathway information were used to generate the network with Cytoscape [33,34].

## 3. Results

### 3.1. Characterization of miRNA in Whole Plasma, EVs and EV-Depleted Plasma 

Approximately 8 million raw reads were obtained in whole plasma, EVs and EV-depleted plasma samples (Table 1). Adapter trimming and removal of short (<15 nt) or low-quality reads resulted in an average of 5 million reads/sample in whole plasma, 4.2 million in EVs and 4.8 million in EV-depleted plasma (Table 1). No significant differences in raw and trimmed read counts were observed between samples from MMD patients and controls. On average, the number of reads mapped to miRNAs was highest in whole plasma samples and lowest in EVs. EV fractions also had the lowest number of observed miRNA (having at least one mapped read without any nucleotide mismatch) and detectable miRNA (having at least 10 mapped reads). Among the detectable miRNAs, 168 are in common in all three sample types (whole plasma, EVs and EV-depleted plasma) from MMD patients and controls (Figure S1). The overall miRNA profile between whole plasma and EV-depleted plasma is more similar than EVs (Figure S2), which is in agreement with a prior report [35]. The top 10 and top five most abundant miRNAs are similar amongst all three sample types (Table S3).

### 3.2. Circulating miRNAs Associated with MMD

**Figure 1:**
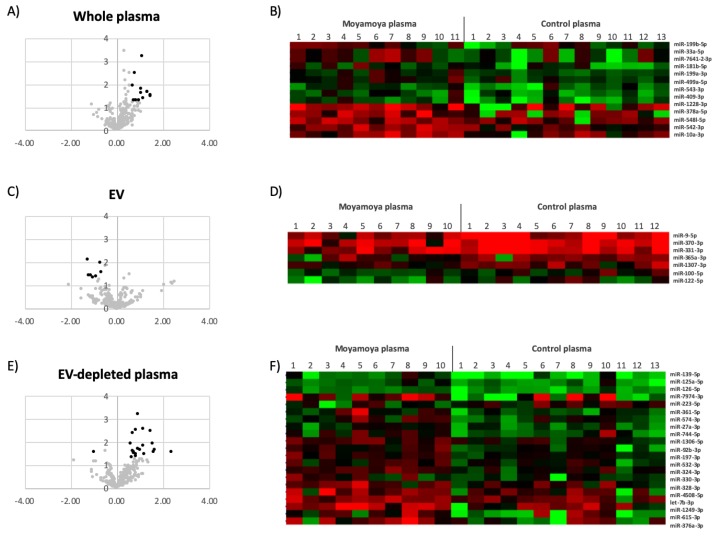
Circulating miRNAs affected by moyamoya. The volcano plots show the effects of moyamoya on circulating miRNA in whole plasma (**A**), EVs (**C**) and EV-depleted plasma (**E**). The black dots in the volcano plots depict that miRNAs were significantly affected. The heat maps are the mean-centered expression profiles of affected miRNAs in whole plasma (**B**), EVs (**D**) and EV-depleted plasma (**F**). The patient conditions are indicated on top of the figures. The colors represent the miRNA concentrations that are either higher (red) or lower (green) than the average concentration of specific miRNA across all the samples.

**Figure 2:**
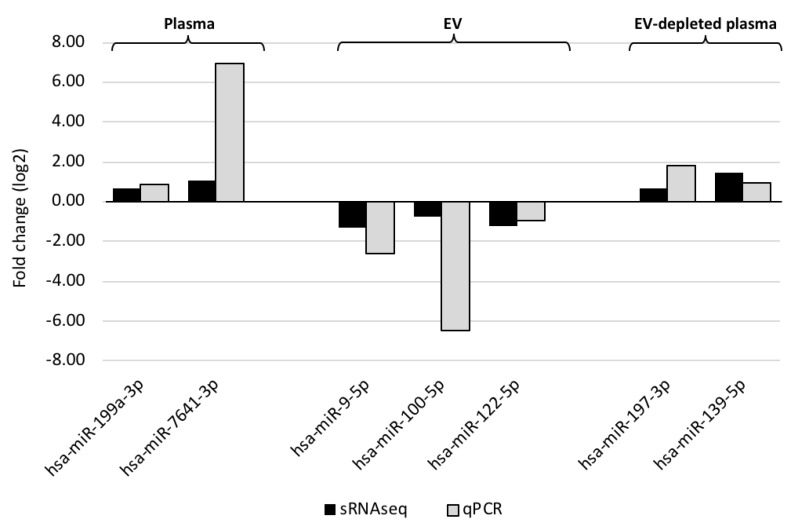
Comparison of miRNA concentration changes measured by qPCR (gray bars) and small RNA sequencing (black bars). The X-axis indicates the identity of affected miRNA, and the Y-axis represents the fold changes in either cycle number (qPCR) or log2-transformed reads per million (RPM)-adjusted small RNAseq read counts. The sample types—whole plasma, EVs and EV-depleted plasma—are indicated on top of the figure.

Even though the overall profiles of miRNA are similar across all samples, a subset of miRNAs showed concentration differences between MMD patients and controls (Figure 1 and Table 2). To assess the accuracy of NGS-based miRNA measurement, the concentration changes of select MMD-affected miRNAs were verified using qPCR (Figure 2). 

The miRNA profiles of EVs and EV-depleted plasma were also compared to determine which miRNA species are preferentially packaged in the vesicles. Of the 176 miRNAs showing significant concentration differences between the two compartments inside of EVs (EV fraction) and outside of EVs (EV-depleted plasma) (>1.5 fold concentration change, *p*-value < 0.05), only 38 had higher concentrations in vesicles (Table S4). MMD status showed no impact on the overall miRNA distribution between inside and outside of EVs. Analyzing the nucleotide composition of these 176 miRNAs revealed an even distribution of four ribonucleotides for miRNAs preferentially located outside of EVs (from EV-depleted plasma) and a higher fraction of “U” and lower fraction of “C” in the EV-encapsulated miRNAs (Figure S3).

### 3.3. Biological Function Associated with MMD-Affected miRNAs

**Figure 3:**
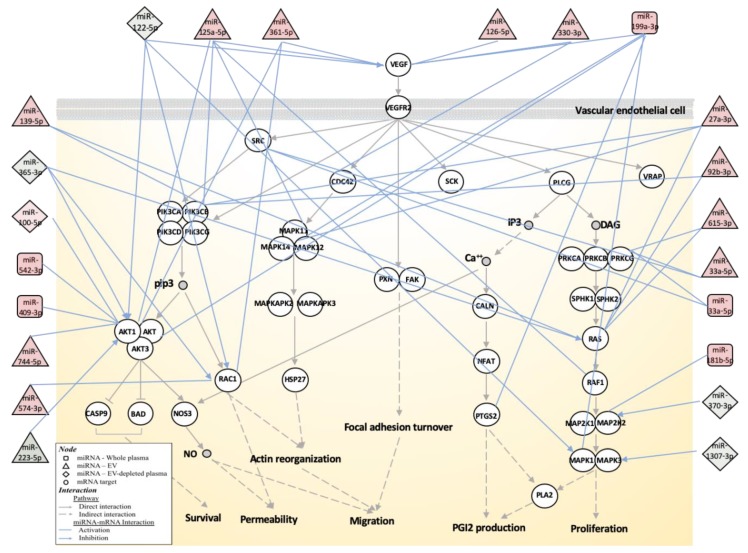
Schematic diagram of mRNA-miRNA interactions associated with the vascular endothelial growth factor (VEGF) signaling pathway. The network is built based on the VEGF signaling pathway map (hsa04370) from the Kyoto Encyclopedia of Genes and Genomes (KEGG), identified by enrichment analysis of miRNA targets. The genes are represented by circles and miRNAs are by squares (whole plasma), triangles (EV) and diamonds (EV-depleted plasma). The identity of genes and miRNAs involved in the process are indicated. The miRNA concentration changes in MMD patients are reflected by either red- (increased concentration in MMD) or green- (decreased concentration in MMD) filled shapes. The miRNA–mRNA interactions are based on validated gene targets from miRTarBase and indicated by light blue lines.

Using mRNA targets demonstrated by at least two different experimental methods (obtained from miRTarBase), the MMD-affected circulating miRNAs may be involved in a number of biological functions including pathways associated with immune response, cell growth and differentiation, signal transduction and nervous system (Table S5). In MMD patients, abnormal angiogenesis and altered levels of angiogenic factors in circulation have been reported [10]. A number of angiogenesis-related biological processes and genes are also targeted by the MMD-affected circulating miRNAs. For example, Angiogenin (*ANG*), a potent mediator of new blood vessel formation, is targeted by miR-409-3p (whole plasma) and miR-223-5p (EV-depleted plasma). The detailed miRNA–mRNA interactions for genes associated with the VEGF pathway are shown in Figure 3.

### 3.4. Impact of MMD on Plasma Proteome 

**Figure 4:**
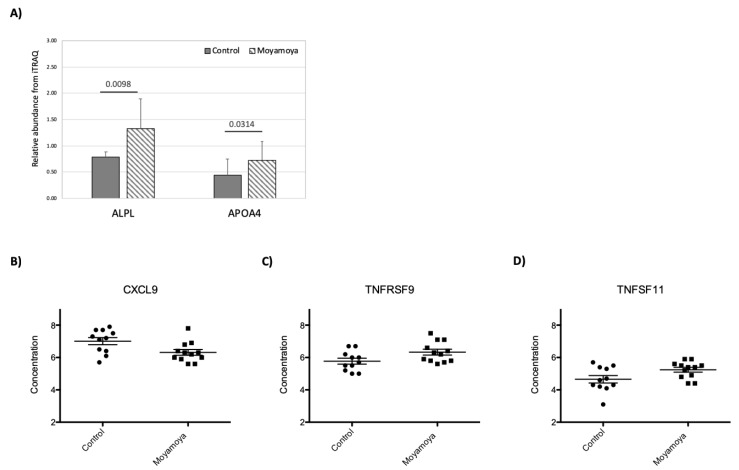
Concentration changes of plasma proteins associated with MMD measured by isobaric tags for relative and absolute quantitation (iTRAQ) (**A**) or proximity extension assay (PEA) (**B**–**D**). For iTRAQ, the protein identities are indicated on the X-axis, the *p*-values and sample groups are indicated on top of the bar graph. For PEA data, sample groups are indicated on the X-axis and the protein abundance is indicated on Y-axis. The protein identity is indicated on top of the figures.

We identified and quantified 529 proteins with iTRAQ. The results showed that MMD has very little impact on the plasma proteome, and only two proteins, Apolipoprotein A IV (APOA4) and Alkaline phosphatase (ALPL) showed statistically significant concentration changes (>1.5 fold concentration difference with a *p*-value < 0.05 based on unpaired simple *t* test) (Figure 4A). We also measured 92 inflammation-related proteins in patient plasma using PEA (Table S2). Comparing MMD patient and control plasma samples, C-X-C motif chemokine 9 (CXCL9), Tumor necrosis factor receptor superfamily member 9 (TNFRSF9), and TNF-related activation-induced cytokine (TNFSF11) showed significant concentration differences (Figure 4B–D). 

### 3.5. RNF213 Mutation Affects Plasma Protein and miRNA Concentrations

Since the mutation status of the *RNF213* gene for MMD patients in this study has been determined (Table S1), we examined the impact of *RNF213* mutation on protein and miRNA profiles in MMD patient plasma. Several miRNAs showed concentration differences associated with the *RNF213* mutations in whole plasma, EVs and EV-depleted plasma (Table 3). Yet hsa-miR-574-3p in EV-depleted plasma is the only miRNA that was also identified as a MMD-affected miRNA (Table 2). Even though the list of *RNF213* mutation-affected miRNAs are different from MMD-affected miRNAs, the pathways targeted by these two sets of miRNAs are similar (Table S6). We did not detect any enriched pathways from *RNF213* mutation-associated miRNAs in whole plasma. One of the controls (individual #10-2) actually is a carrier of the p.R4810K mutation; however, we did not observe the individual’s blood protein and circulating miRNA profiles showed higher similarity to MMD patients including patients, SN10 and SN10-4, within the family.

**Figure 5:**
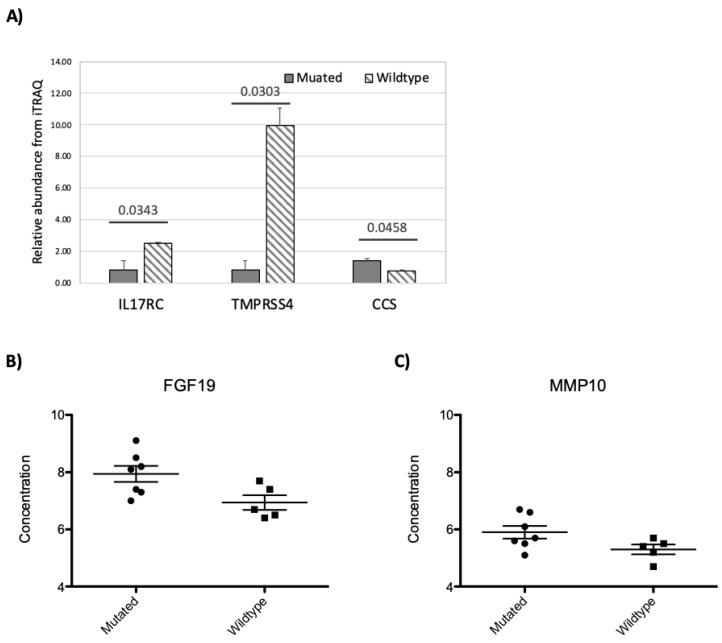
The impact of *RNF213* mutation on plasma proteome measured by iTRAQ (**A**) or PEA (**B**,**C**). The bar graph (iTRAQ) scatter plots (PEA) of proteins showing concentration changes associated with *RNF213* mutation. For iTRAQ (**A**), the protein IDs are indicated on the X-axis, the *p*-values and sample groups are indicated on top of the bar graph. For PEA data (**B** and **C**), sample groups are indicated on the X-axis and the protein abundance is indicated on Y-axis. The protein identity is indicated on top of the figures.

On the plasma proteome, three plasma proteins, interleukin-17 receptor C (IL17RC), transmembrane protease serine 4 (TMPRSS4), and copper chaperone for superoxide dismutase (CCS) showed significant concentration differences between MMD patients with and without a *RNF213* mutation based on the iTRAQ data (Figure 5A). From the PEA results, FGF19 and MMP10 showed significant concentration changes associated with *RNF213* mutation status (Figure 5B,C). 

## 4. Discussion

MMD is a rare inheritable disease with vascular pathologies. Mutations in several genes, including *RNF213* and *ACTA2*, have been linked to the disease [6,8]. The *RNF213* transcript encodes a C3HC4-type Zn-finger protein with ATPase and E3 ligase activities [36]; it targets filamin A (FLNA) and Nuclear Factor of Activated T Cells 2 (NFATC2) to attenuate non-canonical Wnt signaling during vascular development [37]. It may also regulate angiogenic factors, including several MMPs, and endothelial cell proliferation [10,38]. Seven out of 12 patients who participated in this study have an *RNF213* mutation. Since vascular pathologies are the hallmarks of MMD, we conducted comprehensive analyses to assess the impact of MMD and *RNF213* mutation status on the profiles of plasma protein and circulating miRNA. Since RNAs in plasma are “packaged” in two major compartments, lipid vesicle encapsulated and protein complexed, in addition to whole plasma, we also characterized the RNA in EVs (vesicle encapsulated) from plasma, and EV-depleted plasma (outside of EV protein-associated RNAs). Several MMD-associated miRNAs in whole plasma, EVs and EV-depleted plasma were identified. Interestingly, there are few overlapping miRNAs with prior studies on moyamoya-associated circulating miRNA changes [39,40]. This difference was probably caused by different sample types, measurement platforms and NGS library construction methods used in this study. In a study characterizing MMD plasma samples by microarray and qPCR, the authors validated two up-regulated miRNAs, hsa-miR-6722-3p and hsa-miR-328-3p [40]. Our sequencing analysis did not detect miR-6722-3p, but miR-328-3p showed an increased concentration in EV-depleted plasma from MMD patients (Table 2). 

Reports have shown changes to the circulating miRNA spectrum after ischemic stroke [41]. Since one of the major symptoms for patients with MMD is ischemia, the MMD-affected circulating miRNAs may share commonalities with miRNAs associated with ischemic stroke. Indeed, there are some MMD-affected miRNAs, such as miR-27a-3p, that have also been observed in patients with ischemic stroke [42]. Although MMD is a large artery disease [43], the vascular events caused by MMD are usually lacunes in deeply located arterioles in basal ganglia and other subcortical regions. Similar to MMD, cerebral small vessel disease (CSVD) is also characterized by lacunes in deeply located arterioles, in basal ganglia and other subcortical regions. In a recent study, phosphodiesterase 3 (PDE3) was found to play a role in endothelial function and vascular integrity, which was associated with the susceptibility of CSVD. Furthermore, the miR-27a-3p significantly reduced the PDE3 level [44]. 

Levels of miR-9-5p and miR-124-3p, two brain-enriched miRNAs, are decreased in patients with acute ischemic stroke [45]. We have also observed a decrease in miR-9-5p level in EVs from MMD patients. The overall sequence reads mapped to miR-124-3p in our NGS results were too low to obtain reliable data. Another NGS study using CSF from patients with small artery occlusion showed a significant increase in miR-9-5p compared to controls [46]. The opposing direction of concentration change between CSF and EVs may be a result of the blood–brain barrier blocking miRNA complexes from entering the blood stream. 

Changes to the plasma proteome in MMD patients were assessed by both iTRAQ and PEA. The PEA dataset revealed a decrease in CXCL9 and increase in TNFSF11 and TNFRSF9 plasma concentrations in patients with MMD. The three proteins are all involved in T-cell trafficking and activation. CXCL9 is a T-cell chemoattractant and closely related to CXCL10 and CXCL11—both of which showed concentration decreases in MMD patients but did not meet the statistical filtering for MMD-affected proteins (>1.5 fold concentration changes, *p*-value < 0.05). TNFSF11 and TNFRSF9 proteins are members of TNF and TNF-receptor families and are involved in T-cell development and activation. The changes of T-cell function-related proteins suggest the possible involvement of T-cell immunity in MMD pathologies [6,47]. We did not observe significant concentration changes between MMD and control plasma samples in the angiogenic proteins included in the PEA panel. Using validated miRNA–mRNA interactions from miRTarBase [30,31], we did not observe connections between MMD-affected circulating miRNAs and plasma proteins. This is not surprising since the protein and miRNA in circulation may come from different cell types and/or organs in the body.

More than 50% of our MMD patients (seven out of 12) carry a *RNF213* mutation (Table S1). Recent reports have demonstrated the association of p.R4810K with the susceptibility of high blood pressure and coronary heart diseases (CHD) [48,49,50]. Our blood proteome and miRNA analyses identified several *RNF213* mutation-associated changes; for example, the increase in the concentration of a heart-enriched miRNA miR-499a-5p in MMD patient plasma (Table 2). 

Our study is the first to analyze the changes of circulating miRNA inside and outside of EVs associated with MMD. The miRNA profiling results showed a striking difference in the spectrum of MMD-associated miRNAs between inside and outside of EVs. All EV-encapsulated MMD-affected miRNAs showed a decreased concentration. In contrast, the concentrations of all the miRNAs except miR-223-5p were increased in EV-depleted plasma. Even though most of the molecular processes associated with affected miRNAs are similar in the three groups, the *p*-values are lower in EVs. 

Analyzing the miRNA in both EVs and EV-depleted plasma showed that there are more miRNAs outside of EVs (in EV-depleted plasma) and the overall profile of miRNA is different between the two compartments. There are several known processes for cells to sort miRNAs into EVs, especially for exosomes. These include processes mediated by neural sphingomyelinase 2 (nSMase2) protein [51], AGO2 protein [52], miRNAs with uridine (U) at 3’ end [53], and a sequence motif, GGAG, recognized by sumoylated heterogeneous nuclear ribonucleoproteins (hnRNPs) [54]. It is unclear whether the nSMase 2- and AGO2-mediated miRNA sorting are based on specific sequence motif(s). Examining the miRNA sequences between EVs and EV-depleted fractions from our data failed to identify common sequence motif(s) including the reported exosomal miRNA-associated motif GGAG [54]. Since the small RNA sequencing libraries were constructed with adapters containing four degenerated nucleotides (4Ns) at ends, it is difficult for us to examine the non-template addition of nucleotide(s) at the 3’ end of miRNA. However, the miRNAs showing higher concentrations in EVs have a higher percent of U compared to the EV-depleted fraction (Figure S3), which is similar to our prior study on pre-term birth patient samples [26]. This suggests that some miRNAs showed higher concentrations in EVs and these miRNAs are preferentially packaged into EVs by processes yet to be determined. 

In conclusion, we provide results from the first systematic analysis on the impact of MMD and *RNF213* mutations on the profiles of cell-free miRNA and protein in patient plasma samples. The levels of selected MMD-affected miRNAs in the whole plasma, EVs, and EV-depleted plasma have been confirmed by real-time qPCR. We showed that EV-encapsulated miRNA may better reflect the pathology and could provide a noninvasive source of biomarkers to assess the progression of MMD. However, due to the rareness of the disease, the number of patients available for this study and the lack of validation cohort limits the applicability of our findings. Even though we characterized the impact of *RNF213* mutation status on miRNA and protein in circulation, due to the large *RNF213* gene, patients might also carry mutations other than the p.R4810K which might also impact the miRNA and protein profiles in plasma. This study used family member as a control, and this is not ideal since age difference may have impact on the miRNA and protein profiles. However, using family member as a control can significantly reduce the contribution of genetic and environmental factors, since those are known to contribute to various cerebrovascular diseases. Due to unwillingness, Magnetic Resonance Angiography (MRA) was not performed on all unaffected family members, which also limits the application of MMD-associated miRNAs and proteins. A study with a larger patient cohort with a well-characterized mutation status for the *RNF213* gene and proper controls is critically needed to validate the findings and assess its potential applications. 

## Figures and Tables

**Table 1:** Summary of small RNA sequencing results.

Category (mean)	Whole Plasma		Extracellular Vesicle (EV)		EV-Depleted Plasma	
	Moyamoya	Control	Moyamoya	Control	Moyamoya	Control
Raw read count	8,275,547	8,843,102	7,771,542	8,907,214	8,160,659	7,942,143
Trimmed read count	4,962,493	5,229,659	4,134,795	4,165,114	4,232,272	5,505,643
Mapped reads	686,951	672,717	64,844	68,817	191,987	497,845
Observed miRNA (at least one mapped read)	598	574	384	366	467	497
Detected miRNA (at least 10 mapped reads)	333	333	225	233	234	276

**Table 2:** List of moyamoya-affected miRNAs.

Sample Type	miRNA ID	Moyamoya/Control	
		Concentration Change (log 2)	*p*-Value
**Whole Plasma**	hsa-miR-10a-3p	1.12	0.039138
	hsa-miR-1228-3p	1.44	0.034762
	hsa-miR-181b-5p	0.76	0.003383
	hsa-miR-199a-3p	0.70	0.011899
	hsa-miR-199b-5p	0.80	0.049975
	hsa-miR-33a-5p	1.32	0.022562
	hsa-miR-378a-5p	1.47	0.030569
	hsa-miR-409-3p	1.03	0.016035
	hsa-miR-499a-5p	0.95	0.049400
	hsa-miR-542-3p	1.09	0.000614
	hsa-miR-543-3p	0.73	0.049719
	hsa-miR-548l-5p	0.84	0.049396
	hsa-miR-7641-3p	1.06	0.024955
**EV**	hsa-miR-100-5p	−0.73	0.009984
	hsa-miR-122-5p	−1.21	0.037025
	hsa-miR-1307-3p	−0.70	0.027819
	hsa-miR-331-3p	−0.91	0.042423
	hsa-miR-365a-3p	−1.03	0.044760
	hsa-miR-370-3p	−1.13	0.036237
	hsa-miR-9-5p	−1.28	0.007390
**EV-Depleted Plasma**	hsa-let-7b-3p	0.71	0.032790
	hsa-miR-1249-3p	1.53	0.012610
	hsa-miR-125a-5p	0.71	0.024496
	hsa-miR-126-5p	0.60	0.012774
	hsa-miR-1306-5p	0.82	0.003148
	hsa-miR-139-5p	1.47	0.003326
	hsa-miR-197-3p	0.69	0.004247
	hsa-miR-27a-3p	0.74	0.028641
	hsa-miR-324-3p	0.90	0.000670
	hsa-miR-328-3p	0.81	0.036318
	hsa-miR-33a-5p	2.06	0.022378
	hsa-miR-330-3p	0.85	0.045555
	hsa-miR-361-5p	1.12	0.014715
	hsa-miR-376a-3p	1.64	0.023245
	hsa-miR-4508-5p	1.21	0.034114
	hsa-miR-532-3p	0.64	0.048844
	hsa-miR-574-3p	0.93	0.020531
	hsa-miR-615-3p	1.59	0.027183
	hsa-miR-744-5p	1.01	0.023024
	hsa-miR-7974-3p	2.37	0.028771
	hsa-miR-92b-3p	1.13	0.002922
	hsa-miR-223-5p	−1.01	0.027949

**Table 3:** miRNAs showing concentration affected by *RNF213* mutations among MMD patients.

Sample Type	miRNA ID	Mutated/Wildtype	
		Concentration Difference (log 2)	*p*-Value
**Whole Plasma**	hsa-miR-15b-5p	0.68	0.021039
	hsa-miR-17-3p	0.66	0.023532
	hsa-miR-101-5p	0.74	0.006891
	hsa-miR-576-5p	0.70	0.027591
	hsa-miR-766-3p	1.28	0.000960
	hsa-miR-628-3p	−0.88	0.036486
**EV**	hsa-miR-25-3p	0.93	0.034067
	hsa-miR-145-5p	2.35	0.018981
	hsa-miR-148a-3p	0.76	0.024250
	hsa-miR-186-5p	0.70	0.033598
	hsa-miR-188-5p	0.96	0.044339
	hsa-miR-200c-3p	0.89	0.043478
	hsa-miR-330-3p	0.71	0.001653
	hsa-miR-423-3p	1.04	0.033586
	hsa-miR-484-5p	0.78	0.032059
	hsa-miR-486-5p	0.83	0.042868
	hsa-miR-652-3p	0.94	0.022147
	hsa-miR-144-3p	−0.94	0.003342
	hsa-miR-190a-5p	−1.45	0.031924
**EV-Depleted Plasma**	hsa-miR-22-3p	0.76	0.008363
	hsa-miR-320b-3p	0.94	0.009862
	hsa-miR-7641-3p	2.70	0.027610
	hsa-miR-30a-5p	−0.68	0.016327
	hsa-miR-125b-5p	−1.03	0.017852
	hsa-miR-224-5p	−1.43	0.004563
	hsa-miR-421-3p	−1.85	0.047318
	hsa-miR-433-3p	−2.43	0.046585
	hsa-miR-4532-5p	−2.45	0.049418
	hsa-miR-574-3p	−0.92	0.048682

## Supplementary Materials

The following are available online at https://www.mdpi.com/2077-0383/8/10/1648/s1, Figure S1. Venn diagram on number of the detectable miRNA, Figure S2. Correlation of all samples, Figure S3: The sequence composition differences between miRNAs enriched inside and outside of EVs, Table S1: Demographic and clinical phenotype data for patients included in this study, Table S2: List of inflammatory response-related proteins measured by Olink assay, Table S3: List of the top 10 most abundant miRNA , Table S4: List of miRNA showing concentration differences between inside and outside of EVs, Table S5: Biological function associated with moyamoya-affected circulating miRNAs, Table S6: Biological function associated with *RNF213* mutation-affected circulating miRNAs.
