# Supplementary material for: The Impact of Moyamoya Disease and RNF213 Mutations on the Spectrum of Plasma Protein and MicroRNA
Source: J Clin Med. 2019 Oct 10;8(10):1648. doi: 10.3390/jcm8101648 (PMC6832561; doi:10.3390/jcm8101648)

Table S1. Demographic and clinical phenotype data for patients included in the study.

	Sample ID	Age	Sex	Family history	RNF213 Mutation	Measurement			Clinical manifestations	Brain images	Findings from Angiography or MRA
						NGS	PEA	iTRAQ			
Patient with a RNF213 mutation	SN17	17	F	-	p.A1622V	X	X	X	Acute onset weakness of the right side of limbs, sensory deficits on the right side of body. Bilateral EDAS has been performed with improvement of neurological deficits.	No focal lesion on brain parenchyma.	Nearly total occlusion of bilateral proximal MCAs and ACAs with puff of smoke like collaterals.
	SN34	7	M	+	p.V3933M	X	X	X	Insidious onset right hand weakness followed by transient numbness on the right upper limb. Bilateral EDAS with duroplexy have been performed.	Late subacute infarctions at the left fronto-parietal lobe and basal ganglion.	Stenosis of the left ICA and proximal MCA (M1 segment). Occlusion of the left ACA (A1 segment) with collateral circulation of multiple lenticulostriate and thalamoperforating arteries. Collaterals through the left olfactory bulb are also noted.
	SN03	20	F	+	p.R4131C	X	X	X	Acute onset right hemiparesis with gait difficulties. Recurrent headache occurred before the attack. Bilateral EDAS have been performed.	A few old lacunar infarcts on the right basal ganglia.	Severe narrowing at bilateral distal ICA and proximal MCA and ACA. Prominent collaterals at lenticulostriate arteries and leptomeningeal collaterals (from PCA to MCA and ACA). Prominent retrograde flow from bilateral olfactory bulbs to ACA territories.
	SN09	11	M	-	p.R4810K	X	X	X	Acute onset left leg weakness. A few episodes of left hand numbness occurred with total recover of the sensory deficits. Bilateral EDAS has been performed.	No focal lesion on brain parenchyma.	Nearly total occlusion of bilateral supraclinoid ICAs, MCAs and ACAs with puff of smoke like collaterals. Prominent bilateral anterior choroid arteries and P-coms, compatible with moyamoya phenomenon.
	SN25	14	F	-	p.R4810K	X	X	X	Acute onset right hemiparesis with totally recover. Post bilateral STA-MCA anastomosis and the left EDAS have been performed.	No focal lesion on brain parenchyma.	Severe narrowing of bilateral ICAs, MCAs and ACAs with prominent collaterals at lenticulostriate regions and

											collaterals from PCAs to ACA and MCA territories.
	SN10	7	M	+	p.R4810K	X	X		Insidious onset attention deficit, acute onset unsteady gaits and involuntary movement on the left side limbs followed by weakness. The left EDAS on the left side and the indirect EC-IC bypass surgery with pericranium pedical flap on the right one have been performed.	A few old infarctions on the right medial parietal lobe, fronto-parietal and left temporal regions.	Occlusions on bilateral ICA, proximal MCA and ACA. Engorged enlargement of bilateral middle meningeal arteries and superficial temporal arteries.
	SN10-4	18	F	+	p.R4810K		X		Acute onset left hemiparesis. No operation for the cerebral arteries.	No focal lesion on brain parenchyma.	Mildly thin flow signals within proximal bilateral MCAs and ACAs.
Patient without RNF213 mutation	SN11	12	F	-	-	X		X	Acute onset intermittent dizziness, headache, convulsion of left hand and forearm. Intermittent numbness also found in the left hand. Bilateral EDAS has been performed.	No lesion in the brain parenchyma.	Total occlusion of bilateral supraclinoid ICAs, ACAs, and MCAs; increased leticulostriate collaterals. The ACA and MCA territories are supplied by collaterals from ophthalmic arteries, PCA, MMA.
	SN15	14	M	-	-	X	X	X	Acute onset transient dyspraxic aphasia with totally recover. Bilateral EDAS has been performed without significant improvement of collateral circulation. Medical history of hypertension and renal artery stenosis.	No abnormal focal signal change in the brain parenchyma.	Nearly total occlusion of bilateral MCAs (M1 segments) with increased collaterals at basal ganglia regions.
	SN19	20	F	-	-	X	X	X	Acute onset migraine headache on bilateral temporal region. Dizziness with unsteadiness also found. She developed acute onset numbness on the left hand. Bilateral EDAS has been performed.	No abnormal focal signal change in the brain parenchyma.	Moderate to severe stenosis at bilateral ICA bifurcation and bilateral proximal MCAs and ACAs with well-developed collaterals from bilateral lenticulostriate arteries and PCAs.
	SN24	16	M	-	-	X	X	X	Acute onset headache without neurological deficits. Bilateral EDAS has been performed.	Small areas of impaired perfusion at bilateral	Nearly total occlusion of bilateral supraclinoid ICAs and proximal MCAs and ACAs with puff of smoke like collaterals. Prominent

										periventricular white matter.	bilateral anterior choroid arteries, compatible with moyamoya phenomenon.
	SN26	9	F	-	-	X	X	X	Epilepsy with right side weakness after attack. Acute onset left side weakness and facial twitching followed by total recover. Bilateral EDAS has been performed.	A few focal old infarctions at the left frontal white matter.	Total occlusion of bilateral ACAs and bilateral MCAs. Formation of multiple tiny collateral vessels is found at both sides.
Normal Controls	11-1	36	M	-	-	X	X		Unremarkable	N.A.	N.A.
	11-2	35	F	-	-	X	X		Unremarkable	N.A.	N.A.
	11-3	17	M	-	-	X	X		Unremarkable	N.A.	N.A.
	15-1	51	M	-	-	X	X		Unremarkable	N.A.	N.A.
	15-2	48	F	-	-	X	X		Unremarkable	N.A.	N.A.
	15-3	15	F	-	-	X	X		Unremarkable	N.A.	N.A.
	10-1	47	M	-	-	X	X		Unremarkable	N.A.	N.A.
	10-2	44	F	+	p.R4810K	X	X		Unremarkable	No lesion in brain	Normal
	10-3	14	F	-	-	X	X	X	Unremarkable	N.A.	N.A.
	18-3	19	F	-	-	X	X	X	Unremarkable	N.A.	N.A.
	31-3	15	M	-	-	X	X	X	Unremarkable	N.A.	N.A.
	36-3	15	M	-	-	X	X	X	Unremarkable	N.A.	N.A.

ACA = anterior cerebral artery, EDAS = encephaloduroangiosynotosis, F = female, ICA = internal carotid artery, M = male, MCA = middle cerebral artery, MMA = middle meningeal artery, MRA = magnetic resonance image of angiography, PCA = posterior cerebral artery, STA = superficial temporal artery.

Table S2. List of inflammatory response related proteins measured by Olink assay.

Olink assay #	Name used in Olink panel	Official Gene symbol	Description
194	ADA	ADA	Adenosine Deaminase
160	ARTN	ARTN	Artemin
118	AXIN1	AXIN1	Axin-1
103	BDNF	BDNF	Brain-derived neurotrophic factor
184	CASP-8	CASP8	Caspase 8
137	CCL11	CCL11	Eotaxin-1
136	MCP-4	CCL13	Monocyte chemotactic protein 4
145	CCL19	CCL19	C-C motif chemokine 19
115	MCP-1	CCL2	Monocyte chemotactic protein 1
190	CCL20	CCL20	C-C motif chemokine 20
164	CCL23	CCL23	C-C motif chemokine 23
185	CCL25	CCL25	C-C motif chemokine 25
173	CCL28	CCL28	C-C motif chemokine 28
166	MIP-1 alpha	CCL3	Macrophage inflammatory protein 1-alpha
130	CCL4	CCL4	C-C motif chemokine 4
105	MCP-3	CCL7	Monocyte chemotactic protein 3
183	MCP-2	CCL8	Monocyte chemotactic protein 2
108	CD244	CD244	Natural killer cell receptor 2B4
152	PD-L1	CD274	Programmed cell death 1 ligand 1
176	CD40	CD40	CD40L receptor
165	CD5	CD5	T-cell surface glycoprotein CD5
131	CD6	CD6	T cell surface glycoprotein CD6 isoform
107	CDCP1	CDCP1	CUB domain-containing protein 1
196	CSF-1	CSF1	Macrophage colony-stimulating factor 1
123	CST5	CST5	Cystatin D
186	CX3CL1	CX3CL1	Fractalkine
128	CXCL1	CXCL1	C-X-C motif chemokine 1
169	CXCL10	CXCL10	C-X-C motif chemokine 10
117	CXCL11	CXCL11	C-X-C motif chemokine 11
154	CXCL5	CXCL5	C-X-C motif chemokine 5
168	CXCL6	CXCL6	C-X-C motif chemokine 6
101	IL-8	CXCL8	Interleukin-8
122	CXCL9	CXCL9	C-X-C motif chemokine 9
174	DNER	DNER	Delta and Notch-like epidermal growth factor-related receptor
170	4E-BP1	EIF4EBP1	Eukaryotic translation initiation factor 4E-binding protein 1
179	FGF-19	FGF19	Fibroblast growth factor 19
144	FGF-21	FGF21	Fibroblast growth factor 21
139	FGF-23	FGF23	Fibroblast growth factor 23
141	FGF-5	FGF5	Fibroblast growth factor 5
167	Flt3L	FLT3LG	Fms-related tyrosine kinase 3 ligand
106	GDNF	GDNF	Glial cell line-derived neurotrophic factor
156	HGF	HGF	Hepatocyte growth factor
178	IFN-gamma	IFNG	Interferon gamma
162	IL-10	IL10	Interleukin-10
140	IL-10RA	IL10RA	Interleukin-10 receptor subunit alpha
149	IL-10RB	IL10RB	Interleukin-10 receptor subunit beta
157	IL-12B	IL12B	Interleukin-12 subunit beta
159	IL-13	IL13	Interleukin-13
148	IL-15RA	IL15RA	Interleukin-15 receptor subunit alpha
116	IL-17A	IL17A	Interleukin-17A
114	IL-17C	IL17C	Interleukin-17C
133	IL-18	IL18	Interleukin-18

151	IL-18R1	IL18R1	Interleukin-18 receptor 1
125	IL-1 alpha	IL1A	Interleukin-1 alpha
127	IL-2	IL2	Interleukin-2
171	IL-20	IL20	Interleukin-20
121	IL-20RA	IL20RA	Interleukin-20 receptor subunit alpha
150	IL-22 RA1	IL22RA1	Interleukin-22 receptor subunit alpha-1
158	IL-24	IL24	Interleukin-24
124	IL-2RB	IL2RB	Interleukin-2 receptor subunit beta
177	IL-33	IL33	Interleukin-33
180	IL-4	IL4	Interleukin-4
193	IL-5	IL5	Interleukin-5
113	IL-6	IL6	Interleukin-6
109	IL-7	IL7	Interleukin-7
132	SCF	KITLG	Stem cell factor
181	LIF	LIF	Leukemia inhibitory factor
143	LIF-R	LIFR	Leukemia inhibitory factor receptor
195	TNFB	LTA	TNF-beta, Lymphotoxin Alpha
142	MMP-1	MMP1	Matrix metalloproteinase-1
161	MMP-10	MMP10	Matrix metalloproteinase-10
153	Beta-NGF	NGF	Beta-nerve growth factor
182	NRTN	NRTN	Neurturin
188	NT-3	NTF3	Neurotrophin-3
126	OSM	OSM	Oncostatin-M
112	uPA	PLAU	Urokinase-type plasminogen activator
175	EN-RAGE	S100A12	Protein S100-A12
172	SIRT2	SIRT2	SIR2-like protein 2
134	SLAMF1	SLAMF1	Signaling lymphocytic activation molecule
192	STAMPB	STAMBP	STAM-binding protein
191	ST1A1	SULT1A1	Sulfotransferase 1A1
135	TGF-alpha	TGFA	Transforming growth factor alpha
111	LAP TGF-beta-1	TGFB1	Latency-associated peptide transforming growth factor beta 1
163	TNF	TNF	Tumor necrosis factor
110	OPG	TNFRSF11B	Osteoprotegerin
187	TNFRSF9	TNFRSF9	Tumor necrosis factor receptor superfamily member 9
120	TRAIL	TNFSF10	TNF-related apoptosis-inducing ligand
155	TRANCE	TNFSF11	TNF-related activation-induced cytokine
189	TWEAK	TNFSF12	Tumor necrosis factor (Ligand) superfamily, member 12
138	TNFSF14	TNFSF14	Tumor necrosis factor ligand superfamily member 14
129	TSLP	TSLP	Thymic stromal lymphopoietin
102	VEGF-A	VEGFA	Vascular endothelial growth factor A

Table S3. List of the top 10 most abundant miRNA (miRNAs are listed in order of abundance) identified in the different sample types and conditions.

Whole plasma*		EV*		EV-depleted plasma*	
Moyamoya	Control	Moyamoya	Control	Moyamoya	Control
miR-451a-5p	miR-451a-5p	miR-451a-5p	miR-451a-5p	miR-451a-5p	miR-451a-5p
miR-92a-3p	miR-92a-3p	miR-92a-3p	miR-92a-3p	miR-92a-3p	miR-92a-3p
miR-486-5p	miR-486-5p	miR-126-3p	miR-486-5p	miR-486-5p	miR-486-5p
miR-16-5p	miR-16-5p	miR-486-5p	miR-126-3p	miR-16-5p	miR-16-5p
miR-21-5p	miR-21-5p	miR-16-5p	miR-21-5p	miR-21-5p	miR-21-5p
miR-23a-3p	miR-25-3p	miR-21-5p	miR-16-5p	miR-423-5p	miR-423-5p
miR-25-3p	miR-23a-3p	miR-23a-3p	miR-23a-3p	miR-320a-3p	miR-25-3p
miR-423-5p	miR-423-5p	miR-26a-5p	miR-26a-5p	miR-25-3p	miR-320a-3p
miR-320a-3p	miR-22-3p	miR-223-3p	let-7a-5p	miR-23a-3p	miR-19b-3p
miR-22-3p	miR-320a-3p	miR-126-5p	miR-126-5p	miR-22-3p	miR-22-3p

*: The miRNA observed in all three different sample types are listed in boldface characters.

Table S4. List of miRNA showing concentration difference between inside and outside of EV.

	miRNA ID	Sequence	Concentration difference EV-depleted plasma/EV			p-Value		
			All sample	Moyamoya sample	Control sample	All sample	Moyamoya sample	Control sample
Higher concentration inside of EV	hsa-let-7a-1-5p	TGAGGTAGTAGGTTGTATAGTT	-1.06	-1.50	-0.70	8.15E-04	1.18E-03	1.09E-01
	hsa-let-7c-5p	TGAGGTAGTAGGTTGTATGGTT	-1.83	-2.49	-1.32	2.37E-04	6.19E-03	1.35E-02
	hsa-let-7e-5p	TGAGGTAGGAGGTTGTATAGTT	-1.71	-1.76	-1.68	9.69E-08	5.11E-04	8.82E-05
	hsa-let-7f-1-5p	TGAGGTAGTAGATTGTATAGTT	-1.00	-1.57	-0.55	2.21E-03	7.66E-04	2.28E-01
	hsa-let-7f-2-5p	TGAGGTAGTAGATTGTATAGTT	-1.18	-1.74	-0.74	5.96E-04	1.38E-03	1.23E-01
	hsa-miR-103b-1-5p	TCATAGCCCTGTACAATGCTGCT	-0.72	-0.60	-0.82	2.04E-02	1.99E-01	6.66E-02
	hsa-miR-125a-5p	TCCCTGAGACCCTTTAACCTGTGA	-1.12	-1.18	-1.07	3.08E-04	2.52E-02	6.48E-03
	hsa-miR-126-3p	TCGTACCGTGAGTAATAATGCG	-2.96	-3.20	-2.76	1.31E-11	8.17E-07	7.01E-06
	hsa-miR-126-5p	CATTATTACTTTTGGTACGCG	-1.28	-1.42	-1.17	4.90E-05	1.62E-03	1.07E-02
	hsa-miR-132-5p	ACCGTGGCTTTCGATTGTTACT	-0.83	-0.80	-0.84	1.21E-02	1.32E-01	6.24E-02
	hsa-miR-139-5p	TCTACAGTGCACGTGTCTCCAGT	-1.83	-1.54	-2.05	1.70E-07	4.55E-03	8.62E-06
	hsa-miR-142-3p	TGTAGTGTTTCCTACTTTATGGA	-2.13	-2.36	-1.94	1.21E-08	1.03E-04	7.88E-05
	hsa-miR-144-5p	GGATATCATCATATACTGTAAG	-1.07	-1.51	-0.73	4.73E-04	2.63E-03	5.38E-02
	hsa-miR-146b-5p	TGAGAACTGAATTCCATAGGCT	-0.97	-1.79	-0.32	1.66E-02	2.72E-03	5.62E-01
	hsa-miR-150-5p	TCTCCCAACCCTTGTACCAGTG	-1.77	-1.87	-1.68	1.90E-05	4.49E-03	2.61E-03
	hsa-miR-155-5p	TTAATGCTAATCGTGATAGGGGT	-1.31	-1.04	-1.51	1.80E-03	1.38E-01	4.62E-03
	hsa-miR-181c-5p	AACATTCAACCTGTCGGTGAGT	-0.61	-0.81	-0.46	4.24E-02	7.54E-02	2.76E-01
	hsa-miR-190a-5p	TGATATGTTTGATATATTAGGT	-1.12	-1.51	-0.80	2.43E-04	6.36E-03	2.02E-02
	hsa-miR-195-5p	TAGCAGCACAGAAATATTGGC	-1.06	-1.07	-1.04	2.31E-03	5.95E-02	2.01E-02
	hsa-miR-200b-3p	TAATACTGCCTGGTAATGATGA	-0.72	-0.52	-0.87	1.11E-02	2.48E-01	2.26E-02
	hsa-miR-203a-3p	GTGAAATGTTTAGGACCACTAG	-1.44	-1.24	-1.59	9.45E-05	1.05E-02	4.48E-03
	hsa-miR-23b-3p	ATCACATTGCCAGGGATTACC	-1.87	-1.93	-1.82	1.71E-07	1.17E-03	8.84E-05
	hsa-miR-26a-1-5p	TTCAAGTAATCCAGGATAGGCT	-2.05	-2.54	-1.66	2.35E-06	1.31E-05	8.58E-03
	hsa-miR-26b-5p	TTCAAGTAATTCAGGATAGGT	-1.58	-2.11	-1.16	2.02E-05	9.80E-05	2.42E-02
	hsa-miR-28-5p	AAGGAGCTCACAGTCTATTGAG	-0.88	-0.90	-0.88	9.09E-03	5.13E-02	7.47E-02
	hsa-miR-3168-5p	GAGTTCTACAGTCAGAC	-0.87	-0.85	-0.89	6.52E-03	9.71E-02	3.90E-02
	hsa-miR-331-3p	GCCCCTGGGCCTATCCTAGAA	-1.00	-0.76	-1.20	1.85E-03	1.76E-01	1.91E-03
	hsa-miR-338-3p	TCCAGCATCAGTGATTTTGTTG	-1.03	-1.39	-0.75	2.57E-03	1.12E-02	1.01E-01
	hsa-miR-340-3p	TCCGTCTCAGTTACTTTATAGC	-0.99	-1.02	-0.97	3.33E-03	5.55E-02	4.16E-02
	hsa-miR-370-3p	GCCTGCTGGGGTGGAACCTGGT	-0.61	-0.22	-0.94	7.33E-02	6.94E-01	3.47E-02
	hsa-miR-374a-5p	TTATAATACAACCTGATAAGTG	-1.52	-2.01	-1.12	9.74E-04	3.34E-03	7.60E-02
	hsa-miR-374b-5p	ATATAATACAACCTGCTAAGTG	-1.53	-1.84	-1.30	1.98E-05	6.59E-04	7.90E-03
	hsa-miR-378e-3p	ACTGGACTTGGAGTCAGGA	-0.68	-0.67	-0.69	4.36E-02	2.37E-01	1.22E-01
	hsa-miR-411-5p	TAGTAGACCGTATAGCGTACG	-0.85	-0.73	-0.95	3.91E-02	2.36E-01	1.05E-01
	hsa-miR-449a-5p	TGGCAGTGTATTGTTAGCTGGT	-0.55	-1.06	-0.14	6.46E-02	1.26E-02	7.54E-01
	hsa-miR-454-3p	TAGTGCAATATTGCTTATAGGGT	-1.15	-1.41	-0.95	3.71E-04	2.30E-03	3.69E-02
	hsa-miR-615-3p	TCCGAGCCTGGGTCTCCCTCTT	-0.90	-0.65	-1.08	9.69E-03	1.69E-01	3.33E-02
	hsa-miR-6756-5p	AGGGTGGGGCTGGAGGTGGGGCT	-0.53	-0.17	-0.81	5.96E-02	7.25E-01	1.86E-02
Higher concentration outside of EV	hsa-let-7a-1-3p	CTATACAATCTACTGTCTTTC	1.32	1.23	1.39	1.21E-05	6.84E-03	9.33E-04
	hsa-let-7b-3p	CTATACAACCTACTGCCTTCCC	2.60	2.58	2.63	4.15E-15	8.31E-07	4.79E-09
	hsa-let-7b-5p	TGAGGTAGTAGGTTGTGTGGTT	1.03	0.46	1.48	2.71E-04	1.58E-01	7.39E-04
	hsa-let-7d-3p	CTATACGACCTGCTGCCTTTCT	2.49	2.32	2.62	3.80E-13	1.36E-04	5.43E-11
	hsa-let-7d-5p	AGAGGTAGTAGGTTGCATAGTT	0.67	0.09	1.13	1.26E-02	7.94E-01	3.74E-03
	hsa-let-7i-3p	CTGCGCAAGCTACTGCCTTGCT	1.02	0.85	1.16	5.79E-04	7.65E-02	3.60E-03
	hsa-let-7i-5p	TGAGGTAGTAGTTTGTGCTGTT	2.00	1.19	2.64	1.45E-07	1.19E-02	3.30E-06
	hsa-miR-100-5p	AACCCGTAGATCCGAACTTGTG	2.06	1.96	2.13	1.02E-08	4.64E-04	1.04E-05
	hsa-miR-101-1-3p	TACAGTACTGTGATAACTGAA	2.15	1.58	2.59	3.91E-09	1.18E-03	1.76E-06
	hsa-miR-101-1-5p	CAGTTATCACAGTGCTGATGCT	0.62	0.56	0.67	1.67E-02	1.21E-01	8.21E-02
	hsa-miR-103a-1-3p	AGCAGCATTGTACAGGGCTATGA	1.36	0.64	1.94	3.83E-05	1.65E-01	3.29E-05
	hsa-miR-106b-5p	TAAAGTGCTGACAGTGCAGAT	2.01	1.34	2.54	1.07E-07	1.16E-02	3.26E-06
	hsa-miR-10a-3p	CAAATTCGTATCTAGGGGAATA	0.91	0.86	0.93	2.65E-04	2.45E-02	6.18E-03
	hsa-miR-10b-3p	ACAGATTCGATTCTAGGGGAAT	1.28	1.00	1.49	1.21E-07	8.10E-03	8.30E-07
	hsa-miR-1180-3p	TTTCCGGCTCGCGTGGGTGTGT	1.43	1.29	1.53	1.53E-05	1.82E-02	3.36E-04
	hsa-miR-122-5p	TGGAGTGTGACAATGGTGTTTG	3.07	3.08	3.05	1.25E-10	1.85E-04	3.01E-08
	hsa-miR-1228-5p	GTGGGCGGGGGCAGGTGTGTG	0.64	0.94	0.40	2.99E-02	4.51E-02	3.13E-01
	hsa-miR-1246-5p	AATGGATTTTTGGAGCAGG	0.76	0.74	0.78	1.26E-02	1.66E-01	4.29E-02
	hsa-miR-1249-3p	ACGCCCTTCCCCCCCTTCTTCA	1.00	1.13	0.91	3.01E-05	3.22E-03	2.06E-03
	hsa-miR-125b-1-5p	TCCCTGAGACCCTAACTTGTGA	0.79	0.88	0.72	9.81E-03	1.24E-01	3.70E-02
	hsa-miR-128-1-3p	TCACAGTGAACCGGTCTCTTT	1.18	1.11	1.23	2.27E-04	5.08E-02	1.19E-03
	hsa-miR-1301-3p	TTGCAGCTGCCTGGGAGTGACTTC	0.76	0.82	0.71	7.07E-03	7.28E-02	5.11E-02
	hsa-miR-1306-5p	CCACCTCCCCTGCAAACGTCCA	1.85	2.23	1.54	1.85E-08	3.74E-05	4.58E-04
	hsa-miR-1307-3p	ACTCGGCGTGGCGTCGGTCGTG	0.89	1.02	0.77	7.35E-04	2.79E-02	1.28E-02

hsa-miR-1307-5p	TCGACCGGACCTCGACCGGCT	1.60	1.43	1.73	8.59E-08	4.03E-04	1.09E-04
hsa-miR-130a-3p	CAGTGCAATGTTAAAAGGGCAT	2.21	1.85	2.50	3.95E-13	2.17E-05	2.20E-08
hsa-miR-130b-3p	CAGTGCAATGATGAAAGGGCAT	2.59	2.08	2.99	3.18E-13	1.45E-05	1.07E-08
hsa-miR-140-3p	TACCACAGGGTAGAACCACGG	2.52	2.24	2.73	1.42E-09	4.85E-04	1.45E-06
hsa-miR-142-5p	CATAAAGTAGAAAGCACTACT	0.61	0.11	1.02	5.32E-02	8.12E-01	2.44E-02
hsa-miR-144-3p	TACAGTATAGATGATGTACT	1.89	1.59	2.13	2.15E-06	1.06E-02	8.56E-05
hsa-miR-145-5p	GTCCAGTTTTCCCAGGAATCCCT	3.47	2.89	3.93	1.94E-12	6.11E-04	1.04E-11
hsa-miR-146a-5p	TGAGAACTGAATTCCATGGGTT	1.37	0.81	1.82	1.27E-04	9.52E-02	5.21E-04
hsa-miR-148a-3p	TCAGTGCACTACAGAACTTTGT	2.67	2.13	3.09	2.03E-12	1.48E-04	6.50E-09
hsa-miR-148b-3p	TCAGTGCATCACAGAACTTTGT	2.02	1.34	2.56	3.64E-09	5.52E-03	1.73E-07
hsa-miR-150-3p	CTGGTACAGGCCTGGGGGACAG	1.52	1.90	1.21	2.17E-07	2.44E-05	4.05E-03
hsa-miR-152-3p	TCAGTGCATGACAGAACTTGG	1.09	0.78	1.34	3.76E-04	7.84E-02	2.18E-03
hsa-miR-15a-5p	TAGCAGCACATAATGGTTTGTG	2.92	2.46	3.29	1.22E-14	1.29E-05	9.05E-10
hsa-miR-15b-3p	CGAATCATTATTTGCTGCTCTA	2.23	1.68	2.66	2.64E-09	4.00E-03	5.76E-08
hsa-miR-15b-5p	TAGCAGCACATCATGGTTTACA	1.73	1.29	2.08	3.06E-06	1.61E-02	9.53E-05
hsa-miR-16-1-5p	TAGCAGCACGTAAATATTGGCG	2.49	1.99	2.90	4.26E-10	3.30E-04	8.19E-07
hsa-miR-16-2-3p	CCAATATTACTGTGCTGCTTTA	2.91	2.62	3.14	1.17E-11	2.42E-05	3.69E-07
hsa-miR-17-5p	CAAAGTGCTTACAGTGCAGGTAG	0.80	0.56	1.00	1.45E-02	1.69E-01	4.93E-02
hsa-miR-185-5p	TGGAGAGAAAGGCAGTTCCTGA	2.33	1.52	2.98	6.29E-09	6.37E-03	1.29E-07
hsa-miR-18a-3p	ACTGCCCTAAGTGCTCCTTCTGG	0.81	0.91	0.72	1.98E-02	7.08E-02	1.50E-01
hsa-miR-191-5p	CAACGGAATCCCAAAAGCAGCTG	0.65	0.29	0.95	3.56E-02	5.82E-01	1.31E-02
hsa-miR-192-5p	CTGACCTATGAATTGACAGCC	2.08	1.62	2.43	8.47E-08	7.97E-03	2.32E-06
hsa-miR-193a-5p	TGGGTCTTTGCGGGCGAGATGA	2.14	2.10	2.18	6.38E-09	3.97E-04	1.21E-05
hsa-miR-194-1-5p	TGTAACAGCAACTCCATGTGGA	2.53	2.22	2.76	1.05E-10	1.14E-04	4.26E-07
hsa-miR-197-3p	TTCACCACCTTCTCCACCCAGC	1.36	1.42	1.31	1.62E-06	7.41E-03	2.08E-05
hsa-miR-199a-1-3p	ACAGTAGTCTGCACATTGGTTA	0.92	0.56	1.20	4.83E-03	2.65E-01	6.29E-03
hsa-miR-19a-3p	TGTGCAAATCTATGCAAAACTGA	2.64	2.15	3.03	1.12E-11	1.18E-05	2.07E-07
hsa-miR-19b-1-3p	TGTGCAAATCCATGCAAAACTGA	2.90	2.20	3.45	9.36E-13	6.81E-06	2.34E-08
hsa-miR-20a-5p	TAAAGTGCTTATAGTGCAGGTAG	1.26	0.91	1.53	1.52E-04	4.44E-02	1.81E-03
hsa-miR-20b-5p	CAAAGTGCTCATAGTGCAGGTAG	1.18	0.97	1.34	1.28E-04	5.11E-03	5.96E-03
hsa-miR-21-5p	TAGCTTATCAGACTGATGTTGA	1.69	1.25	2.04	3.27E-07	8.03E-03	1.72E-05
hsa-miR-210-3p	CTGTGCGTGTGACAGCGGCTGA	3.14	2.26	3.84	7.07E-12	3.43E-04	2.54E-09
hsa-miR-2110-5p	TTGGGGAAACGGCCGCTGAGTG	0.73	0.77	0.68	8.22E-04	2.31E-02	2.09E-02
hsa-miR-214-3p	ACAGCAGGCACAGACAGGCAGT	1.84	1.65	2.01	1.06E-06	5.90E-03	1.06E-04
hsa-miR-215-5p	ATGACCTATGAATTGACAGAC	1.26	1.23	1.28	2.43E-05	2.96E-03	3.67E-03
hsa-miR-22-3p	AAGCTGCCAGTTGAAGAACTGT	2.69	2.12	3.14	1.04E-12	1.22E-04	1.46E-09
hsa-miR-22-5p	AGTTCTTCAGTGGCAAGCTTTA	3.02	2.30	3.59	7.19E-14	9.00E-05	4.57E-11
hsa-miR-221-3p	AGCTACATTGTCTGCTGGGTTTC	1.91	1.56	2.18	2.90E-09	1.50E-03	6.14E-07
hsa-miR-222-3p	AGCTACATCTGGCTACTGGGT	1.07	0.76	1.32	7.52E-06	2.95E-02	8.93E-05
hsa-miR-223-5p	CGTGTATTTGACAAGCTGAGTT	0.99	0.23	1.57	3.17E-03	6.13E-01	4.75E-04
hsa-miR-23a-3p	ATCACATTGCCAGGGATTTCC	1.04	0.81	1.23	6.33E-04	9.46E-02	2.66E-03
hsa-miR-23a-5p	GGGGTTCCTGGGGATGGGATTT	0.60	0.30	0.84	2.24E-02	5.01E-01	1.18E-02
hsa-miR-24-1-3p	TGGCTCAGTTCAGCAGGAACAG	2.22	1.82	2.54	7.59E-11	2.94E-04	1.06E-07
hsa-miR-25-3p	CATTGCACTTGTCTCGGTCTGA	2.75	2.07	3.28	9.12E-10	1.08E-03	3.45E-07
hsa-miR-27a-3p	TTCACAGTGGCTAAGTTCCGC	1.16	0.87	1.41	7.84E-04	8.48E-02	3.84E-03
hsa-miR-296-5p	AGGGCCCCCCCTCAATCCTGT	0.93	0.93	0.92	1.13E-03	2.29E-02	2.19E-02
hsa-miR-29a-3p	TAGCACCATCTGAAATCGGTTA	1.40	0.87	1.81	8.71E-06	5.21E-02	4.47E-05
hsa-miR-29c-3p	TAGCACCATTTGAAATCGGTTA	2.27	1.71	2.71	3.90E-10	2.24E-03	1.30E-08
hsa-miR-30d-5p	TGTAAACATCCCCGACTGGAAG	1.73	1.37	2.01	3.42E-07	1.28E-02	5.25E-06
hsa-miR-30e-5p	TGTAAACATCCTTGACTGGAAG	1.67	1.15	2.08	5.33E-07	2.23E-02	3.58E-06
hsa-miR-32-5p	TATTGCACATTACTAAGTTGCA	2.19	2.13	2.23	4.86E-08	9.31E-05	1.87E-04
hsa-miR-3200-3p	CACCTTGCGCTACTCAGGTCTG	2.04	1.86	2.18	3.56E-10	3.55E-05	3.96E-06
hsa-miR-320a-3p	AAAAGCTGGGTTGAGAGGGCGA	2.94	2.54	3.24	2.13E-14	5.13E-06	7.66E-10
hsa-miR-320b-1-3p	AAAAGCTGGGTTGAGAGGGCAA	1.98	1.67	2.23	2.09E-10	2.04E-04	9.31E-07
hsa-miR-324-3p	ACTGCCCCAGGTGCTGCTGG	1.04	1.34	0.79	6.78E-05	1.86E-03	1.73E-02
hsa-miR-328-3p	CTGGCCCTCTCTGCCCTTCCGT	1.65	1.66	1.65	1.18E-06	4.34E-03	9.57E-05
hsa-miR-331-5p	CTAGGTATGGTCCCAGGGATCC	2.34	2.11	2.52	5.68E-13	4.77E-05	2.68E-09
hsa-miR-335-5p	TCAAGAGCAATAACGAAAAATGT	1.96	1.58	2.26	5.98E-08	8.09E-03	5.81E-07
hsa-miR-339-3p	TGAGCGCCTCGACGACAGAGCCG	1.89	1.31	2.34	5.61E-07	3.00E-02	1.62E-06
hsa-miR-339-5p	TCCCTGTCCTCCAGGAGCTCACG	0.66	0.56	0.74	1.54E-02	2.71E-01	1.42E-02
hsa-miR-345-5p	GCTGACTCCTAGTCCAGGGCTC	2.30	1.70	2.77	7.50E-10	2.15E-03	2.56E-07
hsa-miR-361-5p	TTATCAGAATCTCCAGGGGTAC	1.19	1.40	1.04	5.62E-04	2.00E-02	1.27E-02
hsa-miR-3613-5p	TGTTGTACTTTTTTTTTTGTTC	1.02	0.89	1.11	6.66E-04	3.32E-02	1.02E-02
hsa-miR-3615-3p	TCTCTCGGCTCCTCGCGGCTC	2.73	2.20	3.15	7.07E-13	3.31E-04	1.23E-10
hsa-miR-362-3p	AACACACCTATTCAAGGATTCA	1.13	0.74	1.43	2.85E-04	9.40E-02	1.50E-03
hsa-miR-363-3p	AATTGCACGGTATCCATCTGTA	2.07	1.30	2.68	3.48E-07	3.60E-02	9.24E-07
hsa-miR-365a-3p	TAATGCCCCTAAAAATCCTTAT	0.67	1.18	0.26	2.16E-02	2.26E-02	4.52E-01
hsa-miR-369-3p	AATAATACATGGTTGATCTTT	0.53	0.19	0.83	7.37E-02	6.89E-01	3.53E-02
hsa-miR-375-3p	TTTGTTCGTTCGGCTCGCGTGA	4.17	3.87	4.41	1.70E-17	1.38E-07	1.26E-10

hsa-miR-376c-3p	AACATAGAGGAAATTCCACGT	0.66	0.34	0.92	3.80E-02	5.02E-01	3.18E-02
hsa-miR-378a-3p	ACTGGACTTGGAGTCAGAAGGC	2.59	2.04	3.02	2.03E-10	1.23E-03	2.13E-08
hsa-miR-423-3p	AGCTCGGTCTGAGGCCCCTCAGT	1.56	1.16	1.87	2.79E-06	2.63E-02	2.45E-05
hsa-miR-423-5p	TGAGGGGCAGAGAGCGAGACTTT	3.44	2.98	3.80	2.03E-17	1.01E-06	7.89E-12
hsa-miR-424-5p	CAGCAGCAATTCATGTTTTGAA	2.50	2.12	2.82	1.15E-10	2.03E-04	2.56E-07
hsa-miR-425-3p	ATCGGGAATGTCGTGTCCGCCC	0.67	0.46	0.83	1.86E-02	2.90E-01	3.29E-02
hsa-miR-425-5p	AATGACACGATCACTCCCGTTGA	2.08	1.63	2.44	2.03E-08	3.03E-03	2.80E-06
hsa-miR-4286-5p	ACCCCACTCCTGGTACC	0.97	0.79	1.12	5.77E-03	1.34E-01	1.86E-02
hsa-miR-4508-5p	GCGGGGCTGGGCGCGCG	2.43	2.65	2.27	2.48E-10	9.27E-05	5.06E-07
hsa-miR-451a-3p	TAGTAATGGTAATGGTTCTCT	1.22	0.82	1.54	3.24E-06	2.57E-02	8.23E-05
hsa-miR-451a-5p	AAACCGTTACCATTACTGAGTT	2.19	1.61	2.65	7.26E-09	1.04E-03	2.82E-06
hsa-miR-4532-5p	CCCCGGGGAGCCCGGCG	0.71	0.42	0.95	4.58E-02	5.14E-01	1.31E-02
hsa-miR-4732-3p	GCCCTGACCTGTCCTGTTCTG	1.87	1.78	1.94	3.74E-10	7.16E-04	1.02E-07
hsa-miR-4732-5p	TGTAGAGCAGGGAGCAGGAAGCT	1.93	1.90	1.94	8.10E-09	3.94E-04	1.39E-05
hsa-miR-483-3p	TCACTCCTCTCCTCCCGTCTT	1.31	1.24	1.35	1.40E-04	2.89E-02	2.50E-03
hsa-miR-483-5p	AAGACGGGAGGAAAGAAGGGAG	1.57	1.18	1.87	2.16E-05	5.28E-02	1.09E-04
hsa-miR-484-5p	TCAGGCTCAGTCCCCTCCCGAT	3.05	2.68	3.35	1.11E-15	1.03E-05	1.81E-11
hsa-miR-486-1-3p	CGGGGCAGCTCAGTACAGGAT	1.81	1.64	1.94	1.93E-08	1.81E-03	1.11E-05
hsa-miR-486-1-5p	TCCTGTACTGAGCTGCCCCGAG	3.85	3.48	4.13	3.36E-17	8.47E-07	1.49E-11
hsa-miR-497-5p	CAGCAGCACACTGTGGTTTGT	1.10	1.39	0.86	6.57E-04	8.67E-03	3.96E-02
hsa-miR-500a-3p	ATGCACCTGGGCAAGGATTCTG	1.12	0.38	1.70	2.94E-03	5.31E-01	2.90E-04
hsa-miR-501-3p	AATGCACCCGGGCAAGGATTCT	1.50	1.22	1.72	7.66E-07	5.48E-03	2.33E-04
hsa-miR-502-3p	AATGCACCTGGGCAAGGATTCA	1.78	1.36	2.10	1.09E-05	3.92E-03	1.29E-03
hsa-miR-505-3p	CGTCAACACTTGCTGGTTTCCT	1.55	1.10	1.91	2.07E-05	6.68E-02	6.69E-05
hsa-miR-532-3p	CCTCCCACACCCAAGGCTTGCA	1.05	1.04	1.07	1.69E-04	1.62E-02	9.48E-03
hsa-miR-532-5p	CATGCCTTGAGTGTAGGACCGT	1.79	1.48	2.02	5.28E-07	9.08E-03	1.17E-05
hsa-miR-548q-5p	GCTGGTGCAAAAGTAATGGCGG	1.50	1.31	1.64	1.79E-08	4.62E-04	2.23E-05
hsa-miR-550a-1-3p	TGTCTTACTCCCTCAGGCACAT	1.80	1.71	1.87	1.89E-08	2.87E-03	7.97E-07
hsa-miR-550a-1-5p	AGTGCCTGAGGGAGTAAGAG	1.35	1.19	1.47	1.40E-06	1.57E-03	3.96E-04
hsa-miR-576-5p	ATTCTAATTTCTCCACGTCTTT	0.77	0.41	1.06	7.67E-03	3.60E-01	6.57E-03
hsa-miR-584-5p	TTATGGTTTGCCTGGGACTGAG	1.41	0.92	1.79	9.24E-05	3.34E-02	1.49E-03
hsa-miR-590-5p	GAGCTTATTCATAAAAGTGCAG	0.65	0.28	0.94	2.85E-02	5.61E-01	1.36E-02
hsa-miR-598-3p	TACGTCATCGTTGTCATCGTCA	1.10	0.64	1.46	1.03E-04	7.45E-02	5.45E-04
hsa-miR-6087-5p	TGAGGCGGGGGGGCGAGC	0.73	0.60	0.85	3.13E-02	2.65E-01	4.90E-02
hsa-miR-628-3p	TCTAGTAAGAGTGGCAGTCGA	0.91	0.85	0.96	2.43E-03	1.26E-01	4.45E-03
hsa-miR-629-5p	TGGGTTTACGTTGGGAGAACT	1.48	1.25	1.65	1.05E-06	2.45E-03	2.23E-04
hsa-miR-652-3p	AATGGCGCCACTAGGGTTGTG	1.98	1.31	2.52	2.11E-09	6.27E-03	2.22E-08
hsa-miR-660-5p	TACCCATTGCATATCGGAGTTG	2.83	2.45	3.13	1.21E-12	4.45E-05	1.80E-08
hsa-miR-874-3p	CTGCCCTGGCCCGAGGGACCGA	1.30	1.13	1.44	3.85E-04	5.07E-02	4.44E-03
hsa-miR-885-5p	TCCATTACACTACCCTGCCTCT	2.93	2.97	2.89	1.63E-12	1.61E-05	2.00E-07
hsa-miR-92a-1-3p	TATTGCACTTGTCCCGGCCTGT	3.66	3.51	3.77	7.06E-18	2.79E-07	2.78E-11
hsa-miR-92a-2-3p	TATTGCACTTGTCCCGGCCTGT	1.44	1.00	1.79	2.85E-06	3.50E-02	2.85E-05
hsa-miR-92b-3p	TATTGCACTCGTCCCGGCCTCC	1.30	1.66	1.02	3.16E-05	2.22E-03	8.90E-03
hsa-miR-93-5p	CAAAGTGCTGTTCGTGCAGGTAG	1.81	1.13	2.34	2.87E-06	2.98E-02	4.67E-05
hsa-miR-96-5p	TTTGGCACTAGCACATTTTTGCT	1.65	1.75	1.57	6.39E-06	4.50E-04	3.83E-03
hsa-miR-99a-5p	AACCCGTAGATCCGATCTTGTG	1.85	1.58	2.05	1.21E-08	1.77E-03	2.85E-06

Table S5. Biological function associated with Moyamoya affected circulating miRNAs.

Pathway Description			Whole plasma	EV	EV-depleted plasma
Environmental Information Processing	Signal transduction	ErbB signaling pathway	1.10E-07	3.30E-03	1.40E-14
		mTOR signaling pathway	7.20E-06	6.00E-03	1.90E-07
		VEGF signaling pathway	1.50E-05	8.90E-02	2.00E-06
		MAPK signaling pathway	7.90E-04	6.40E-06	1.40E-08
		Wnt signaling pathway	8.10E-04		2.50E-09
		TGF-beta signaling pathway	3.00E-02	1.30E-02	7.40E-09
		Notch signaling pathway		3.90E-03	2.60E-02
		Jak-STAT signaling pathway		2.30E-02	2.20E-06
		Hedgehog signaling pathway			1.50E-02
Cellular Processes	Transport and catabolism	Endocytosis		2.30E-02	2.40E-03
		Autophagy		4.40E-02	
	Cell motility	Regulation of actin cytoskeleton	6.60E-02	5.30E-02	7.50E-05
	Cell growth and death	Apoptosis	7.60E-03	7.10E-04	3.80E-03
		Oocyte meiosis	2.20E-02		
		Cell cycle	3.90E-02	2.20E-02	7.00E-08
		p53 signaling pathway		1.80E-02	6.50E-04
	Cellular community	Focal adhesion	6.80E-16	9.20E-06	2.10E-12
		Adherens junction	1.30E-04	5.00E-05	8.20E-09
		Tight junction	5.10E-02	8.20E-02	
		Gap junction			1.40E-02
Organismal Systems	Immune system	Toll-like receptor signaling pathway	2.90E-05	2.60E-02	2.40E-04
		Fc epsilon RI signaling pathway	1.50E-04	9.90E-02	1.80E-05
		T cell receptor signaling pathway	2.80E-04	2.90E-03	5.80E-06
		B cell receptor signaling pathway	6.90E-04	2.70E-02	3.00E-07
		Leukocyte transendothelial migration	3.00E-02	5.00E-02	1.00E-03
		Fc gamma R-mediated phagocytosis	4.10E-02		2.00E-03
		Chemokine signaling pathway	8.00E-02	9.30E-03	6.60E-07
		Natural killer cell mediated cytotoxicity			8.00E-03
	Endocrine system	Adipocytokine signaling pathway	6.00E-06	1.10E-04	2.10E-05
		Progesterone-mediated oocyte maturation	4.70E-05	6.50E-04	2.30E-04
		Insulin signaling pathway	3.30E-04	8.80E-06	1.20E-06
		GnRH signaling pathway	3.30E-03		7.10E-04
		Melanogenesis	1.40E-02		2.00E-04
		Aldosterone-regulated sodium reabsorption	8.70E-03		5.50E-04
	Nervous system	Neurotrophin signaling pathway	8.50E-07	3.40E-06	5.80E-12
		Long-term depression	1.20E-02		3.70E-02
		Long-term potentiation	4.70E-02		1.10E-02
	Development	Dorso-ventral axis formation	1.30E-02	2.20E-03	1.10E-02
		Axon guidance			6.70E-04

Table S6. Biological function associated with RNF213 mutation affected circulating miRNAs.

Pathway Description			Whole plasma	EV	EV-depleted plasma
Environmental Information Processing	Signal transduction	ErbB signaling pathway		5.30E-07	9.50E-08
		Jak-STAT signaling pathway			1.10E-02
		MAPK signaling pathway		3.40E-05	2.10E-07
		mTOR signaling pathway		4.90E-04	2.00E-04
		Notch signaling pathway		3.90E-02	
		TGF-beta signaling pathway		3.60E-06	9.90E-04
		VEGF signaling pathway		1.90E-04	1.90E-03
		Wnt signaling pathway		8.30E-03	6.70E-02
	Signaling molecules and interaction	Cytokine-cytokine receptor interaction		2.70E-02	9.30E-02
		ECM-receptor interaction		8.10E-02	

Figure S1. Venn diagram on number of the detectable miRNA.

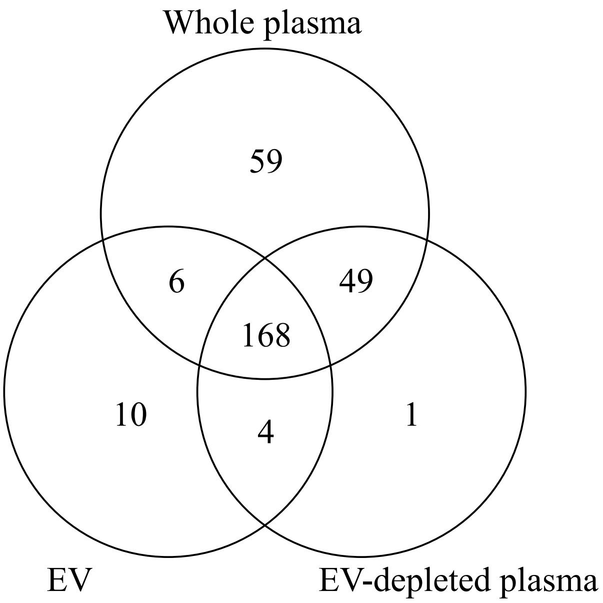

Figure S2. Correlation of all samples.

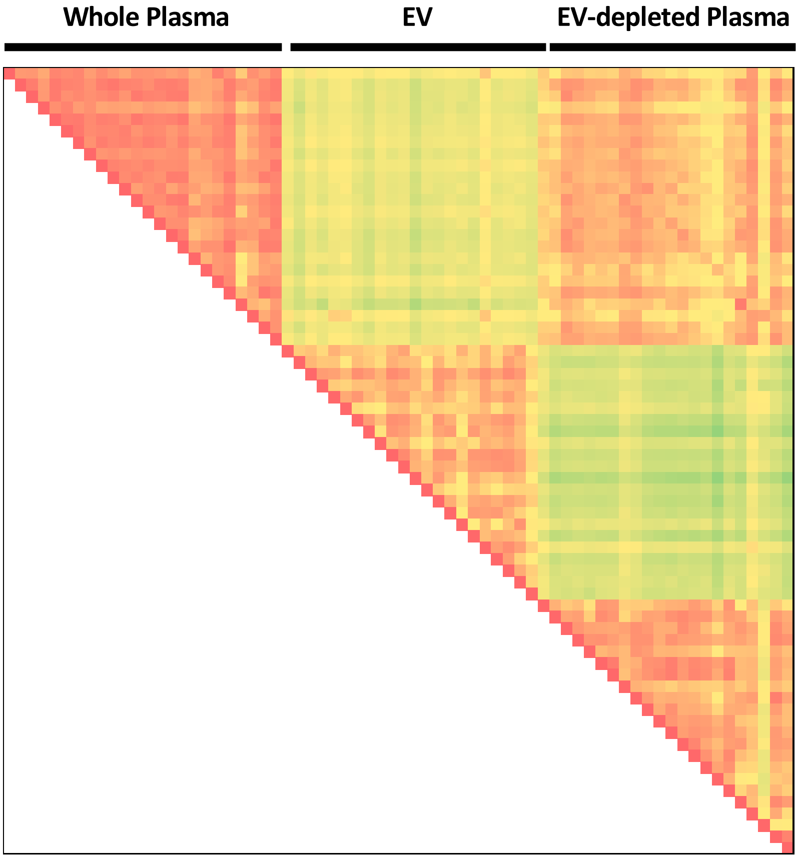

Figure S3. The sequence composition differences between miRNAs enriched inside (black bars) and outside (gray bars) of EVs. The brackets on top of the figure indicate significant difference. X-axis represents the 4 different nucleotides and its fractions among miRNAs enriched either inside or outsides of EV are indicated in Y-axis.

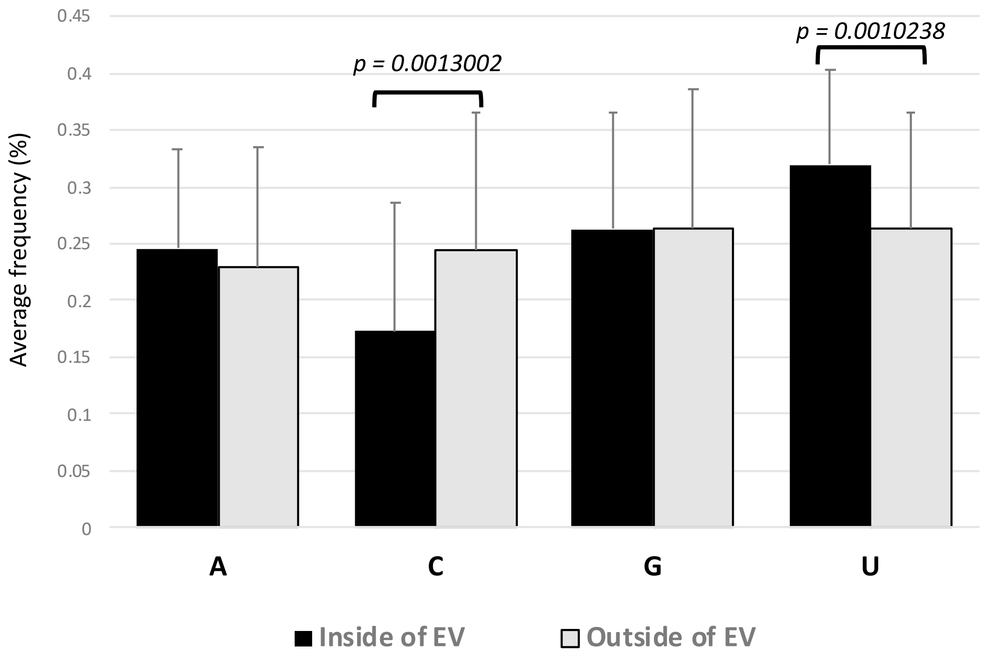

Supplement: Supplementary file 1 [file jcm-08-01648-s001.pdf]
